# The Restoration of Energy Pathways Indicates the Efficacy of Ketamine Treatment in Depression: A Metabolomic Analysis

**DOI:** 10.1111/cns.70324

**Published:** 2025-03-09

**Authors:** Zerui You, Xiaofeng Lan, Chengyu Wang, Haiying Liu, Weicheng Li, Siming Mai, Haiyan Liu, Fan Zhang, Guanxi Liu, Xiaoyu Chen, Yanxiang Ye, Yanling Zhou, Yuping Ning

**Affiliations:** ^1^ Key Laboratory of Neurogenetics and Channelopathies of Guangdong Province and the Ministry of Education of China Guangzhou Medical University Guangzhou China; ^2^ Guangdong Engineering Technology Research Center for Translational Medicine of Mental Disorders Guangzhou China; ^3^ Department of Child and Adolescent Psychiatry The Affiliated Brain Hospital, Guangzhou Medical University Guangzhou China; ^4^ Clinical Laboratory The Affiliated Brain Hospital, Guangzhou Medical University Guangzhou China

**Keywords:** ketamine, major depressive disorder, metabolomic, thyroxine

## Abstract

**Aims:**

Despite the clinical benefits of ketamine in treating major depressive disorder (MDD), some patients exhibit drug resistance, and the intricate mechanisms underlying this await comprehensive explication. We used metabolomics to find biomarkers for ketamine efficacy and uncover its mechanisms of action.

**Methods:**

The study included 40 MDD patients treated with ketamine in the discovery cohort and 24 patients in the validation cohort. Serum samples from the discovery cohort receiving ketamine were analyzed using ultra performance liquid chromatography‐mass spectrometry to study metabolomic changes and identify potential biomarkers. Metabolic alterations were evaluated pre‐ and post‐ketamine treatment. Spearman correlation was applied to examine the relationship between metabolite alterations and depressive symptom changes. In addition, potential biomarkers, particularly thyroxine, were investigated through quantitative measurements in the validation cohort.

**Results:**

We found that energy metabolite changes (adenosine triphosphate, adenosine diphosphate [ADP], pyruvate) were different in responders versus non‐responders. The magnitude of the ADP shift was strongly correlated with the rate of reduction in Montgomery‐Asberg Depression Rating Scale (MADRS) scores (Rho = 0.48, *p*_FDR_ = 0.018). Additionally, baseline free triiodothyronine (FT3) levels are inversely associated with the rate of MADRS reduction (Rho = −0.645, *p* = 0.017).

**Conclusions:**

Ketamine ameliorates depressive symptoms by modulating metabolic pathways linked to energy metabolism. Low baseline FT3 levels appear to predict a positive response in MDD patients, suggesting FT3 has potential as a biological marker for clinical ketamine treatment.

**Trial Registration:** ChiCTR‐OOC‐17012239

## Graphical abstract

We investigated the metabolic alterations resulting from ketamine administration. Ketamine alters mitochondrial function, increases ATP/ADP, impacts BDNF in responders, and lower baseline FT3 is associated with greater MADRS reduction in ketamine treatment.

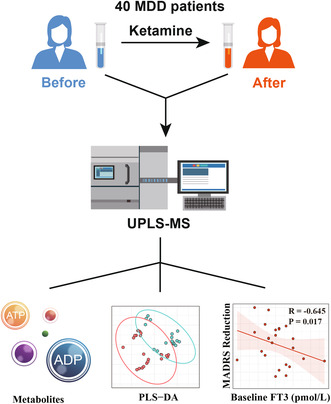

## 1 Introduction

Ketamine has expeditious and robust antidepressant effects, triggering significant theoretical and clinical progress in the treatment of major depressive disorder (MDD) [1]. Ketamine has been used as a rapid‐acting antidepressant for depression patients, while simultaneously diminishing the intensity of suicidal ideation [2, 3, 4, 5]. Previous studies have shown that the antidepressant and anti‐suicidal effects of six repeated ketamine injections can persist for more than two weeks [6, 7]. Despite these proven benefits, approximately 30%–40% of patients fail to show a marked response to this treatment [2, 7]. A meta‐analysis revealed a significant increase in brain‐derived neurotrophic factor (BDNF) levels, observed exclusively in the population responsive to ketamine treatment. However, no appropriate prognostic biomarkers were found to be effective in differentiating therapeutic responses prior to the ketamine treatment [8] and the exact mechanism of ketamine's therapeutic action remains incompletely understood.

Metabolomics has emerged as a valuable research field in psychiatry, offering insights into the mechanisms underlying antidepressant treatment response and the associated metabolic pathways [9, 10]. Previous research has successfully elucidated the roles of plasma metabolites and metabolomic alterations brought about by antidepressants [11, 12]. Acylcarnitines may be a pivotal biochemical mechanism underpinning the clinical heterogeneity of MDD, especially when coalesced with clinical phenotypic profiles [12]. The modulatory effects of selective serotonin reuptake inhibitors (SSRIs) on beta‐oxidation and mitochondrial energetics of acylcarnitines may constitute a potential mechanistic link. Moreover, Caspani and colleagues employed lipoproteins as a predictive tool to determine the therapeutic responses of patients treated with escitalopram or aripiprazole [13]. Metabonomics might have a crucial role in unraveling the agent of the antidepressant and anti‐suicidal effects of ketamine, offering a promising avenue for developing innovative therapeutic strategies for depression. Such endeavors are particularly pressing given the need to untangle the enigmatic mechanisms of ketamine's therapeutic action.

In our previous study, we found that six ketamine infusions, administered repetitively to MDD patients, were safe, swift, and potent, with an efficacy rate approaching 70% [6]. Within the kynurenine metabolic pathway, subjects manifesting a therapeutic response to ketamine evinced remarkably augmented levels of kynurenine (KYN) and kynurenic acid (KYNA)/KYN ratio in comparison to non‐responders, concomitant with concurrent reductions in the Montgomery–Asberg Depression Rating Scale (MADRS) scores [14]. However, probable discrepancies in the activity of other metabolic pathways remain unclear. Identification of such nuances could improve our ability to delineate the therapeutic effects of ketamine, utilizing ultra performance liquid chromatography–tandem mass spectrometry (UPLC‐MS) analysis. The primary objective of our investigation is to discern the pivotal metabolomic pathways that are modulated by ketamine and to discover potential biomarkers that are closely related to its pharmacological effects. Based on the MADRS scores obtained after administering ketamine, the study participants were dichotomized into two groups: responders and non‐responders, and the responders were defined as those exhibiting a reduction of ≥ 50% in the MADRS. We employed metabolomics analysis to ascertain (1) whether responders showed a discernible metabolic profile that was distinct from non‐responders; (2) whether the observed differential metabolites between the responders and non‐responders had the potential to function as robust predictive biomarkers for ketamine administration; and (3) whether the concentrations of the identified differential metabolites were correlated with the alleviation of depressive symptoms. Our goal is to identify potential biomarkers that could predict ketamine's antidepressant efficacy and elucidate the underlying mechanism of action of ketamine.

## 2 Methods

### 2.1 Participants

This investigation was performed as a component of an open‐label, non‐randomized clinical trial (Chinese Clinical Trial Registry, registration number: ChiCTR‐OOC‐17012239), wherein patients presenting with depression were subjected to repeated infusions of ketamine as part of their treatment protocol. Building upon the original experiment, we screened an approximate number of MDD patients in both the responder and non‐responder cohorts. The ethics committees of the Affiliated Brain Hospital of Guangzhou Medical University approved this study. All subjects provided written informed consent.

The inclusion criteria for patients included: (1) aged 18 to 65 years; (2) diagnosed with MDD according to the Diagnostic and Statistical Manual of Mental Disorders, 5th edition; (3) Hamilton Depression Rating Scale (HAMD‐17) scores ≥ 17 [15]; (4) treatment resistance, which is characterized by a lack of response to two or more antidepressant treatment trials with full dosage and duration, or with suicide ideation, defined as a Beck Scale for Suicide Ideation (SSI)‐part *I* score ≥ 2 [16]. The comprehensive exclusion criteria have been explicated in our previous study [14]. This study included a discovery cohort of 40 MDD patients and a validation cohort of 24 MDD patients, all derived from the same sample population.

### 2.2 Study Design

All patients in the discovery and validation cohort were scheduled to receive six ketamine infusions (0.5 mg/kg, i.v.) in 50 mL normal saline over a duration of 40 min under supervision by experienced healthcare professionals for 12 days (day 1, 3, 5, 8, 10, and 12). All patients maintained a consistent dosage of their prescribed antidepressant for at least 4 weeks before and during the study. The fasting plasma samples and clinical assessments were obtained on the day before the first infusion (baseline, T0, day 0) and the day after the sixth infusion (post‐treatment, T1, day 13). Efficacy assessments were measured by the Montgomery–Asberg Depression Rating Scale (MADRS). Response was defined as a reduction of at least 50% in the total score of MADRS compared to the baseline [17]. The percent change in MADRS score was calculated for each patient treated with ketamine as follows: ([{T1—T0}/T0] × 100). Additional design details can be found in our prior publication [6].

### 2.3 Blood Collection and Metabolomics Analysis

We collected fasting blood using 5 mL vacutainer tubes containing heparin lithium, followed by centrifugation of the samples for 12 min at 1147 rcf and 4°C within 1 h, typically within 10–20 min of collection. Afterward, in the discovery cohort, we aliquoted the supernatant into Eppendorf tubes and froze them at −80°C until performing ultra performance liquid chromatography‐tandem mass spectrometry (UPLC‐MS) analysis. A detailed description of the methods used for sample preparation, data acquisition, and processing for UPLC‐MS analysis, as well as metabolite identification, can be found in the Data S1. In the validation cohort, thyroid function tests were performed via a commercial chemiluminescence assay on an Alinity i system (Thyroid function tests assay, Abbott Laboratories, Abbott Park, IL, USA). Thyroid function tests (TFTs) included free triiodothyronine (FT3), free thyroxine (FT4), total triiodothyronine (TT3), total thyroxine (TT4), and thyroid‐stimulating hormone (TSH).

### 2.4 Statistical Analysis

We removed metabolites with greater than 80% missing ratios from the sample. Missing values were imputed with the minimum value. After logarithmic (base 2) transformation and scaling [18], principal component analysis (PCA) and partial least square discriminant analysis (PLS‐DA) were performed on all the endogenous metabolites [19]. PCA is an unsupervised method for dimension reduction that clearly shows the within‐group repeatability and between‐group differences, helping to assess reproducibility and detect outliers. In contrast, PLS‐DA, a supervised method, maximizes group separation and provides information on the importance of features for distinguishing groups. According to the PLS‐DA model, variable importance in the projection (VIP) values were assessed for every metabolite, which described their ability to discriminate between groups. Metabolites with VIP values greater than 1 were selected for subsequent analysis with Welch's unpaired *t*‐test at the univariate level [20]. Non‐parametric statistical tests were utilized, including the Mann–Whitney *U* test and the Wilcoxon signed‐rank test. *P*‐values were adjusted for multiple testing using the Benjamini–Hochberg procedure [21].

For the evaluation of metabolites' diagnostic performance and preferable presentation [22, 23], after log_2_ transformation, the receiver operating characteristic curve (ROC) analysis and expression of the metabolites were calculated. ROC curves were used to assess the predictive capacity of baseline differential metabolites between groups for the antidepressant effect of ketamine. The area under the ROC curve (AUC), 95% confidence intervals (CI), sensitivity, and specificity values were used to gauge the diagnostic performance of metabolites.

Correlations between the change in metabolite expression ([{T1—T0}/T0] × 100) and the percentage change of the MADRS score were calculated using Spearman correlation [24]. For testing whether metabolic profiles were different among groups before and after ketamine treatment, linear mixed models were developed [25]. Fixed effects included group categories (responders or non‐responders), time point (T0 or T1), group categories × time point, sex, age, BMI, and baseline MADRS score. Random effects included a random intercept for each individual. Sex, age, BMI, and baseline MADRS score are commonly taken into account as covariates. Partial correlations were used to investigate the relationship between metabolites and MADRS reduction. Furthermore, additional factors including treatment duration, age of onset, antidepressant dosages, smoking status, and alcohol consumption are taken into consideration to evaluate the stability of the findings. Dosages of all antidepressants were converted into fluoxetine equivalents using established literature, and in cases where multiple agents were administered daily, their total dose was computed.

Integrated pathway analysis was carried out by MetaboAnalyst 5.0 (https://www.metaboanalyst.ca) for performing biological function. Continuous data are presented as means ± standard deviation (SD) or median with interquartile range (IQR). Normality tests were performed using Shapiro–Wilk normality tests. Unless otherwise stated, statistical analyses were performed in R (R version 4.3.1). Analysis of variance (ANOVA), Mann–Whitney *U*, or Chi‐squared tests were used to compare the clinical characteristics between responders and nonresponders using SPSS software (version 25; SPSS Inc., Chicago, IL, USA). The threshold for statistical analyses was set at *p* < 0.05 for all analyses.

### 2.5 Validation

We conducted an external validation study to accurately quantify the levels of thyroxine before ketamine treatment. Thyroid function tests were performed on a total of 24 ketamine‐treated MDD patients who had available serum samples and clinical data at baseline. The validation cohort was compared to discovery cohorts concerning sex, age, and BMI, and was found to be well matched (Tables S1 and S2). In this cohort of patients, 6 individuals did not fully comply with the ketamine treatment. To appraise the efficacy of their post‐treatment outcomes, we utilized the MADRS score 24 h after the final injection.

## 3 Results

### 3.1 Demographics and Clinical Characteristics

In the discovery cohort, twenty‐three patients (57.5%) were categorized as responders and seventeen (42.5%) as non‐responders. There were no significant differences between responders and non‐responders in sex, age, BMI, duration of illness, and baseline MADRS scores (30.78 ± 5.70 vs. 33.18 ± 7.41, *p* = 0.255). Compared with non‐responders, the responders showed significantly lower MADRS scores (*T* = −10.74, *p* < 0.001) after ketamine treatment. During ketamine treatment, both groups used similar types of medication and fluoxetine equivalents (all *p* > 0.05). Additional details are provided in Table 1.

**TABLE 1:** Clinical characteristics of the participants in the discovery cohort.

Characteristic	Total (*n* = 40)	Non‐responser (*n* = 17)	Responser (*n* = 23)	Statistics *p* ^a^
Sex, male, *n* (%)^b^	16 (40.0%)	6 (35.3%)	10 (43.5%)	0.845
Age (years), median (IQR)^c^	33 (24–46)	33 (22–41)	33 (26–46)	0.380
BMI, mean (SD)^d^	21.93 (3.48)	21.78 (3.45)	22.04 (3.58)	0.816
Education (years), median (IQR)^c^	12 (9–15)	12 (9–15)	12 (9–16)	0.515
Onset of age (years), median (IQR)^c^	24 (19–35)	22 (19–34)	25 (19–36)	0.622
Duration (months), median (IQR)^c^	51 (23–111)	48 (12–72)	72 (26–120)	0.366
Smoking, smoker, *n* (%)^b^	5 (12.5%)	2 (11.8%)	3 (13.0%)	1.000
Baseline MADRS score, mean (SD)^d^	21.93 (3.48)	33.18 (7.41)	30.78 (5.70)	0.276
Post‐treatment MADRS score, mean (SD)^d^	14.55 (11.03)	25.47 (7.13)	6.48 (4.31)	**< 0.001**
Current antidepressant medications				
Fluoxetine equivalents, mg, mean (SD)^d^	41.12 (23.30)	36.18 (22.74)	44.78 (23.52)	0.251
Antipsychotics, *n* (%)^b^	27 (67.5%)	14 (82.4%)	13 (56.5%)	0.085
Benodiazepine, *n* (%)^b^	22 (55.0%)	9 (52.9%)	13 (56.5%)	0.822
Emotional stabilizer, *n* (%)^b^	7 (17.5%)	4 (23.5%)	3 (13.0%)	0.388

^a^ The *p*‐value was calculated by comparing nonresponders and responders (*p* < 0.05 in bold)*.*

^b^ Analyzed by the Chi‐squared test.

^c^ Analyzed by Mann–Whitney *U* test.

^d^ Analyzed by Independent two‐sample *t*‐test.

### 3.2 Pre‐Treatment Metabolomic Profiling of Patients

**FIGURE 1:**
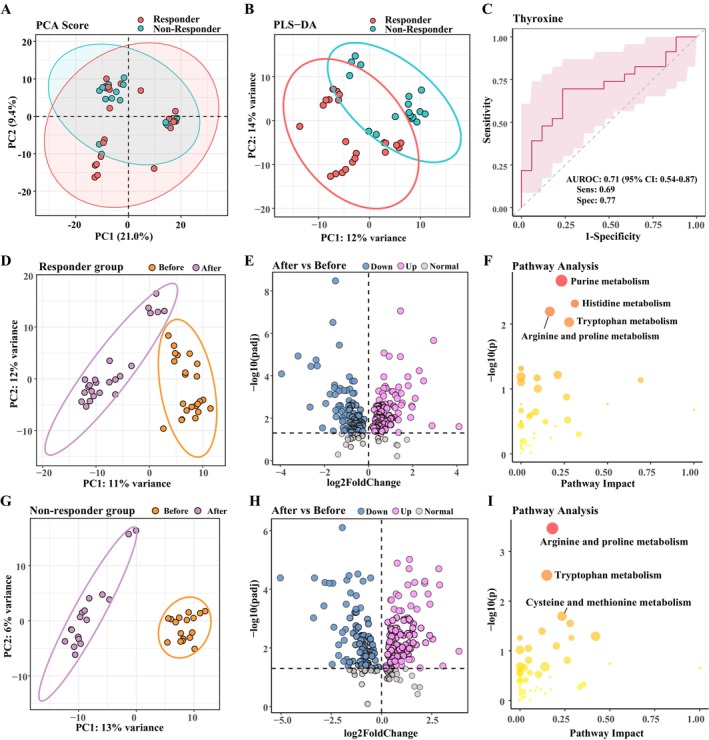
Disparate serum metabolomic profiles pre‐ and post‐treatment among the responders and non‐responders in the discovery cohort. (A, B) PCA (A) and PLS‐DA (B) scatter of serum metabolomics data compared responder and non‐responder group before ketamine treatment. (C) Distinctive characterization of thyroxine prior to treatment. (D–F) PLS‐DA scatter plot (D), volcano plot (E), and differential metabolite pathway analysis (F) for metabolomics data in the ketamine responder group before and after treatment. (G–I) PLS‐DA scatter plot (G), volcano plot (H), and differential metabolite pathway analysis (I) for metabolomics data in the ketamine non‐responder group before and after treatment.

For the untargeted metabolomics analysis in our study, a total of 647 endogenous metabolites were identified and analyzed. After log transformation and scaling, PCA analysis (Figure 1A) was used to display the overall distribution of the metabolomic expression, and the PLS‐DA score plot (Figure 1B) was performed to identify differences in metabolic profiles between responders and non‐responders. In the PLS‐DA score plot, a notable distinction was observed between the two groups.

After filtering with VIP score > 1, differential metabolites were chosen using non‐corrected univariate statistical significance criteria (*p* < 0.05). Thirty‐four metabolites showed significant differences and could distinguish between both groups before treatment. None of these differences were statistically significant after multiple testing correction. Among them, we consider thyroxine to be an appropriate metabolic indicator, which is already widely used in clinical practice, with a significant increase in the responder group before treatment (*T* = 2.42, *p* = 0.020). Responders exhibited pre‐treatment high expression of thyroxine compared to non‐responders and presented with reduced thyroxine after ketamine treatment. ROC analyses were performed to quantify the diagnostic performance. Of the differential metabolites, thyroxine exhibited a promising diagnostic potential with an area under the ROC curve (AUC) of 0.71 (Figure 1C), ranking high among the metabolites tested. The detailed information on these metabolites is presented in Sheet S1.

### 3.3 Ketamine‐Induced Changes Among Responders and Non‐Responders

To detect the variables that distinguish responders and non‐responders, the cut‐offs used were VIP score > 1 and adjusted *p* < 0.05 obtained by Wilcox nonparametric tests. One hundred and eighty‐two metabolites in the responder group and 198 metabolites in the non‐responder group were found to have significantly changed (Figure 1D–I). More information regarding the differential metabolites can be found in Sheets S2 and S3. These distinct changes in the responder group were accompanied by functional alterations in amino acid metabolism, such as purine, histidine, arginine and proline, and tryptophan metabolism during ketamine progression, as depicted in Figure 1F. The differential metabolites in the non‐responder group mainly participated in arginine and proline metabolism, tryptophan metabolism, and cysteine and methionine metabolism (Figure 1I).

### 3.4 Investigation of Metabolite Level Changes and Their Correlation With MADRS Score

**FIGURE 2:**
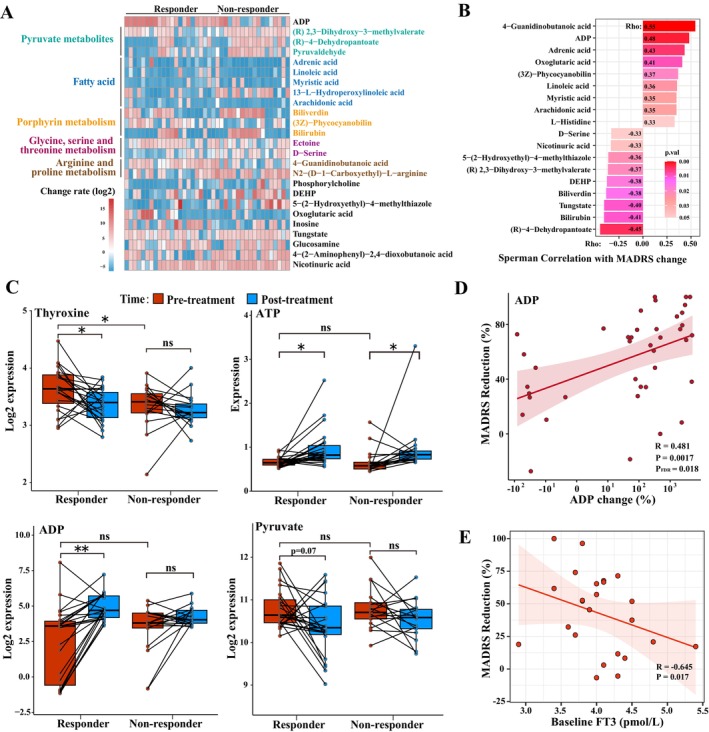
Alterations in important differential metabolite expression between the responders and non‐responders. (A) Heatmap of metabolites alterations between responders and non‐responders before and after ketamine treatment in the discovery cohort. Changes in metabolites, expressed as the log_2_‐transformed rate, are illustrated. Red indicates a significant increase in metabolites after ketamine treatment, while blue denotes a decrease. (B) Spearman correlation of selected metabolites with MADRS score reduction rate. The rectangle color indicates *p*‐value significance: the darker the red, the smaller the *p*‐value, while the length of the rectangle reflects the strength of the correlation. (C) Expression alterations and group difference in important differential metabolite profiles pre‐ and post‐treatment among the two groups. Ketamine groups, including responders and non‐responders, are shown in red before treatment and in blue after treatment. (D) Spearman correlation between the rate of ADP change and the rate of MADRS reduction. (E) The partial correlation between baseline FT3 and MADRS reduction in the validation cohort. **p < 0.01; *p < 0.05. ADP, adenosine diphosphate; ATP, adenosine triphosphate; DEHP, Bis(2‐ethylhexyl) phthalate; FT3, free triiodothyronine.

We analyzed the post‐log_2_ change of each metabolite to detect any noteworthy discrepancies in the alterations occurring before and after therapy. The heatmap illustrates the metabolites exhibiting different rates of change before and after treatment between two groups (Figure 2A, *p* < 0.05). Several long‐chain fatty acids exhibited a reduction after treatment, except for 13‐L‐Hydroperoxylinoleic acid (LOOH), which increased in most nonresponders.

Using a mixed linear model and Spearman's correlation analysis, we screened for metabolites that exhibited significant group‐by‐time interaction effects and were associated with MADRS scores. Eighteen metabolites stand out. These metabolites have clear implications in metabolic pathways, such as pyruvate metabolism [(R)‐2,3‐dihydroxy‐3‐methylvalerate, (R)‐4‐dehydropantoate], energy catabolism (adenosine diphosphate), arginine metabolism (4‐guanidinobutanoic acid), long‐chain fatty acids (adrenic acid, linoleic acid, arachidonic acid, and myristic acid), glycerophospholipid metabolism (phosphorylcholine), histidine metabolism (L‐histidine), and chemical components [bis(2‐ethylhexyl)phthalate, tungstic acid] and so on (Figure 2B, *p* < 0.05). The results remained stable after controlling for more metabolically related covariates, such as antidepressant use, smoking status, and alcohol consumption (Tables S3 and S4).

Our study found that adenosine diphosphate (ADP) and pyruvate‐related metabolites exhibited a strong association with MADRS reduction rates and conspicuous variation in change rates between the two groups (Table S5). Thyroxine, adenosine triphosphate (ATP), ADP, and pyruvate expression maps depicting the dissimilarities between pre‐ and post‐treatment of the two groups are presented in Figure 2C. Moreover, ADP expression change was positively correlated with changes in patients' MADRS scores (Figure 2D, Rho = 0.481, *p*_FDR_ = 0.018). Changes in the rate of thyroxine, which were previously considered a concern, did not exhibit a significant correlation with changes in MADRS scores (Rho = −0.22, *p*_FDR_ > 0.05). In the partial correlation, the associations between the change of ADP and other significant metabolites with changes in MADRS scores were retained while controlling for covariates (Table S6).

### 3.5 External Validation

We aimed to examine whether routine thyroid function tests conducted in hospitals could serve as predictors for the efficacy of ketamine therapy. The external validation cohort was matched to the discovery cohort for sex, age, and BMI. In the validated cohort, the responding and non‐responding groups had similar demographic characteristics and thyroid function expression (Tables S1, S2, and S7). The rate of MADRS reduction during ketamine treatment was negatively correlated with baseline FT3 levels (Rho = −0.645, *p* = 0.017, Figure 2E) in the partial correlation after adjusting for confounding factors. Our targeted approach yielded no meaningful correlations between ketamine response and FT4 or TT4, which diverged from previous non‐targeted metabolomics analyses. Details of thyroid function in the external validation cohort are shown in Table 2. Upon removal of 6 patients who did not complete the ketamine administration (with 5 of them exhibiting negligible response), the partial correlation (Rho = −0.681, *p* = 0.043, Table S8) showed the negative association between initial FT3 levels and MADRS reduction persisted statistically significant.

**TABLE 2:** Assoication of the rate of MADRS score with pre‐treatment thyroid function in the validation cohort by using partial correlation in patients with complete ketamine treatment.

Variables	Model 1^a^		Model 2^b^	
	Cor	*p* value	Cor	*p* value
TSH, mIU/L	0.011	0.963	0.026	0.933
FT3, pmol/L	−0.423	0.063	−0.645	**0.017**
FT4, pmol/L	−0.113	0.635	−0.262	0.388
TT3, nmol/L	−0.244	0.301	−0.385	0.194
TT4, nmol/L	−0.059	0.806	−0.277	0.360

*Note:* The partial correlation was used to assess the association between MADRS reduction and metabolites changes (log_2_‐transformed). Significant values (*p* < 0.05) are in bold.

Abbreviations: FT3, free triiodothyronine; FT4, free thyroxine; TSH, thyroid‐stimulating hormone; TT3, total triiodothyronine; TT4, total thyroxine.

^a^ In model 1, we additionally adjusted for sex, age, BMI, and baseline MADRS score in the partial correlation.

^b^ In model 2, we additionally adjusted for sex, age, BMI, baseline MADRS score, duration, fluoxetine equivalents, smoking status, alcohol consumption, and age of onset in the partial correlation.

## 4 Discussion

To our understanding, the present study represents the initial comprehensive investigation into the metabolic alterations triggered by recurrent ketamine injections in patients with MDD. The current study has revealed several noteworthy findings: (1) preceding the administration of ketamine treatment, no significant disparities in metabolomics were observed between the respondents and non‐respondents; (2) before ketamine injection, the metabolic phenotype of patients exhibited similarities between responder and non‐responder groups, and a lower baseline concentration of thyroid hormone FT3 in patients might indicate susceptibility to ketamine; (3) following ketamine administration, striking differences in metabolite alterations were detected between the two patient groups, supporting an enhanced energy metabolic pathway in the responder group.

Prior studies have hinted at potential relationships between patient response to ketamine and changes in mitochondrial β‐oxidation of fatty acids [26, 27]. This present study elucidates a more intuitive function of ketamine in ameliorating disrupted mitochondrial and energy metabolism in MDD patients. We found that ATP significantly increased in all MDD patients. ADP was positively linked to changes in MADRS scores in all MDD patients but only significantly increased in the responder group. This observed change in ADP, as well as the trend in the ATP/ADP ratio, aligns with findings from previous studies conducted in the mouse hippocampus [28, 29]. While the energetic ratios in our study are consistent with those reported in other research, these findings require cautious interpretation and further validation due to potential influences from sample handling and processing [30].

One of the fundamental mechanisms underlying ketamine's pharmacological effects is the inhibition of *N*‐methyl‐D‐aspartate receptor (NMDAR) activity, subsequently leading to the release of BDNF in neurons [31]. BDNF exerts a critical influence on both the conventional antidepressant response and the rapid antidepressant efficacy that arises from ketamine treatment [32]. Emerging research has highlighted the therapeutic potential of BDNF analogs in mitigating mitochondrial dysfunction and bolstering ATP production [33]. Meanwhile, energy currency compensates for the increased energy demand and cellular energy deficit in activated synapses and increases BDNF expression and neurogenesis, ultimately leading to the mitigation of depressive symptoms and associated oxidative stress [29, 34, 35]. Earlier studies have also reported a substantial contribution of ATP to both the maturation and transportation processes of BDNF [36, 37]. While BDNF was not directly measured in our study, evidence suggested a significant increase in BDNF levels among the patients who exhibited a favorable response to ketamine treatment [8]. Our results suggested that, in the ketamine treatment, the responder subgroup exhibited increased levels of ATP and ADP following treatment, which may be associated with BDNF.

**FIGURE 3:**
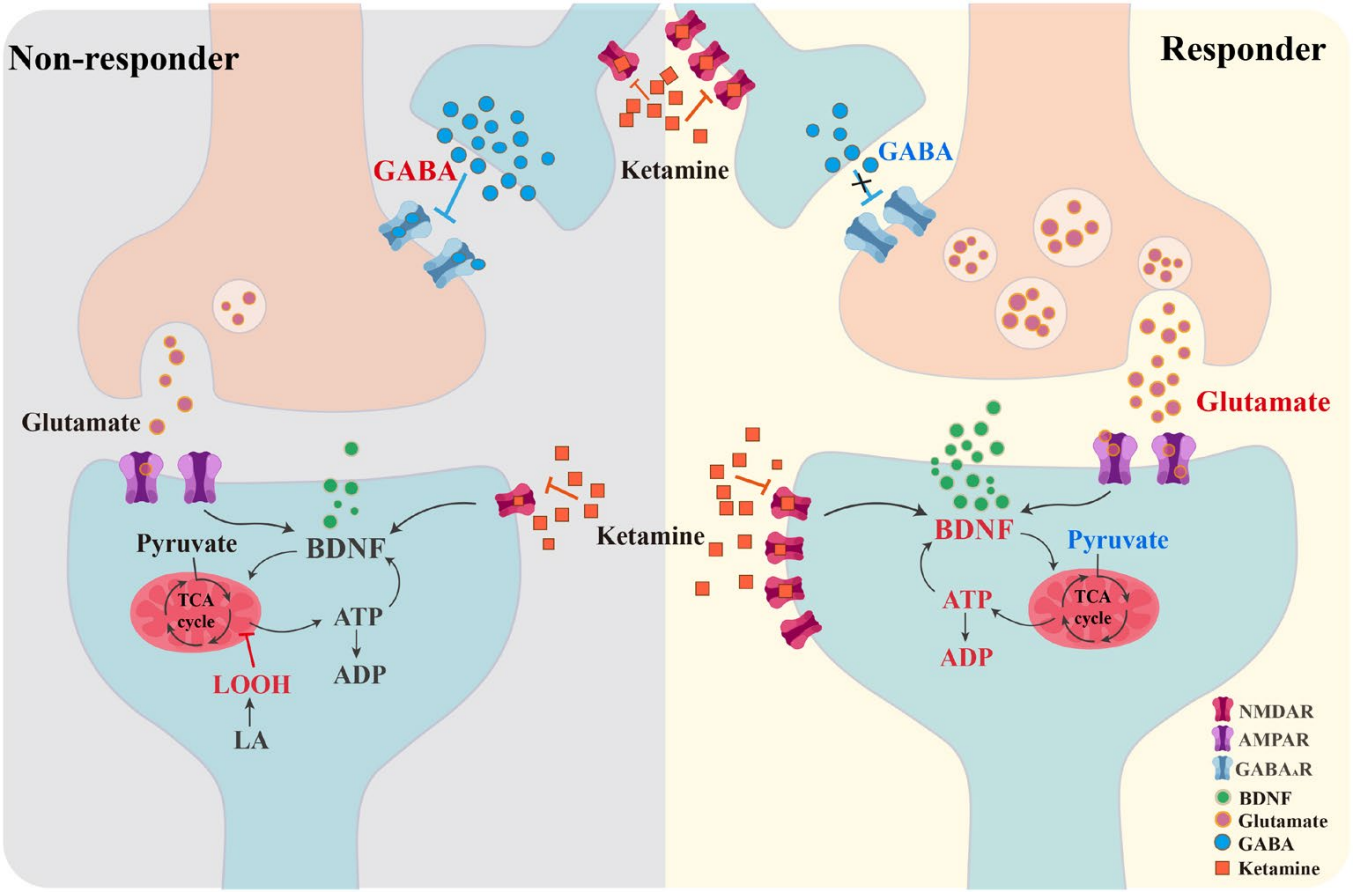
Metabolite changes between two groups: schematic representation. In the diagram, up‐regulated or down‐regulated metabolites are illustrated by the colors red and blue, respectively.

Numerous evidences indicate that the reciprocal facilitation between ATP and BDNF leads to synaptic growth and plasticity mediating the antidepressant effects of ketamine [38], which is more likely to occur in the responder group. Furthermore, an increase in ADP levels, the metabolite of ATP, was positively correlated with greater improvement in MADRS scores, suggesting a potential link with augmented BDNF production. The alterations in the levels of other metabolites were consistent with our hypothesis. For instance, pyruvate, an essential substrate in the tricarboxylic acid (TCA) cycle, exhibited a decreasing trend due to the heightened requirement for energy production. Consequently, this typically led to decreased quantities of byproducts associated with pyruvate metabolic pathways, particularly within the responder groups, including (R)‐4‐dehydrovalerate and (R)‐2,3‐dihydroxy‐3‐methylpentanoate. A graphical representation showcasing the alterations in the differentially expressed metabolites is presented in Figure 3.

Our investigation did not discern a meaningful divergence in the metabolic phenotypes of the responder and non‐responder subgroups before ketamine treatment, indicating that the metabolic distribution among our recruited patients was relatively homogeneous before treatment and that the disparities witnessed between groups after treatment principally arose from the effects of the ketamine injections. Only a few metabolites passed the uncorrected *p*‐value. Among them, thyroxine stood as the most prominent due to its widespread use in general hospitals. Thyroxine plays a crucial role in maintaining normal central nervous system function [39] and can also indicate underlying disease states. Recent studies have demonstrated that MDD patients who have attempted suicide have notable thyroid dysfunction [40, 41, 42], characterized by predominantly lower levels of thyroxine [43].

We observed that the thyroxine concentrations in the cohort exhibiting a response before treatment were elevated relative to the non‐response group and exhibited a notable decline following treatment. However, in external validation, no difference was found in thyroxine between the two groups before treatment. This result may be attributed to the limited sample size in this study.

In the external validation, lower baseline FT3 levels are associated with better treatment outcomes after receiving ketamine treatment. This result indicates that FT3 holds promise as a valuable biomarker for predicting the likelihood of a favorable response to ketamine therapy, taking into account demographic variables when analyzing the data. Wang and colleagues unveiled a robust positive relationship between elevated anxiety levels in patients and increased NMDAR expression, while conversely, a remarkable inverse relationship was observed between the concentration of FT3 and the expression of NMDAR [44]. Ketamine is an ionotropic glutamatergic NMDAR antagonist that produces a prompt antidepressant response in patients with MDD [45, 46]. Thus, it is postulated that MDD patients with lower FT3 concentrations may have more NMDAR expression, thereby potentially elevating the sensitivity of ketamine to brilliant antidepressant outcomes. Per our discernment, thyroxine function emerges as a promising indicator of the potency of ketamine‐based interventions in MDD patients. Additionally, few studies have investigated the mechanism underlying the relationship between ketamine and FT3. The previous study found that high concentrations of ketamine temporarily inhibited the production of FT3, cyclic AMP (cAMP), and thyroglobulin, while FT4 levels increased in the thyroid follicle [47]. We anticipate forthcoming research with larger study cohorts to validate and broaden this promising discovery and the potential mechanisms underlying the interaction between ketamine and thyroid function.

Analogous to earlier investigations, both responder and non‐responder cohorts evinced a conspicuous decline in the rate of metabolite change in specific long‐chain fatty acids following ketamine treatment [26, 27]. However, the non‐responders demonstrate a marked escalation in the rate of metabolism for a specific fatty acid, 13‐L‐Hydroperoxylinoleic acid (LOOH). LOOH, a type of lipid hydroperoxide converted from linoleic acid (LA), is known to substantially heighten the extent of reactive oxygen species (ROS) production, which are implicated in the pathophysiology of depression and the heterogeneity of treatment response [48, 49]. This increase in ROS levels can trigger the mitochondrial membrane potential (MMP), apoptotic nuclear condensation, as well as DNA fragmentation [50, 51], and eventually may lead to impaired ATP production [52, 53]. Under low γ‐aminobutyric acid (GABA) concentrations, LOOH was observed to enhance GABA receptor activity, potentially by augmenting GABA's affinity for the receptor [54]. LOOH might instigate the inhibitory function of interneurons and curtail the efficacy of ketamine [55, 56]. These observations posit that LOOH plays a noteworthy role in GABAergic neurotransmission and might have important implications for the treatment of depression.

Our study exhibits specific restrictions that should be considered. First, the small sample size limits the interpretation of findings. Patients were taking other psychotropic medications concomitantly with ketamine treatment, which was more appropriate for depression in real‐world conditions and improved the applicability of our study [57]. The dynamic nature of metabolic processes and potential variations in sample handling suggest that our findings should be interpreted cautiously [30]. Due to the presence of the blood–brain barrier, peripheral metabolic findings might not fully align with those in the brain. Although BDNF has been identified as a key therapeutic target for ketamine response, we did not examine the expression of BDNF or the correlation between BDNF and FT3 levels. Future research could explore this relationship to enhance understanding of biomarkers for ketamine efficacy. Finally, our study included treatment‐resistant MDD patients and MDD patients with a suicidal mind. Further investigations are required to determine the generalizability of our findings to individual subtypes of depression.

## 5 Conclusions

We have observed that ketamine effectively mitigates symptoms of depression by regulating the metabolic pathways associated with ATP, ADP, and pyruvate. In addition, individuals with lower baseline levels of thyroid hormone FT3 might exhibit greater susceptibility to the response to ketamine. Our findings provide promising indications for the advancement of ketamine treatment in addressing MDD.

## Author Contributions

**Zerui You:** conceptualization, methodology, software, validation, visualization, data curation, writing – original draft, writing – review and editing. **Chengyu Wang, Xiaofeng Lan:** methodology, resources, investigation, writing – review and editing. **Haiying Liu, Weicheng Li, Guanxi Liu, Xiaoyu Chen, Siming Mai, Fan Zhang, Yanxiang Ye, Haiyan Liu:** methodology, resources, investigation. **Yanling Zhou:** conceptualization, funding acquisition, project administration, methodology, writing – review and editing. **Yuping Ning:** conceptualization, funding acquisition, investigation, supervision, project administration.

## Conflicts of Interest

The authors declare no conflicts of interest.

## Supporting information


Data S1.

Data S2.

## Data Availability

The data that support the findings of this study are available from the corresponding author upon reasonable request.
