# Supplementary material for: The Restoration of Energy Pathways Indicates the Efficacy of Ketamine Treatment in Depression: A Metabolomic Analysis
Source: CNS Neurosci Ther. 2025 Mar 9;31(3):e70324. doi: 10.1111/cns.70324 (PMC11890978; doi:10.1111/cns.70324)
Supplement: Supplementary file 2 — Data S2. [file CNS-31-e70324-s002.docx]

**[Supplementary Materials](D:/u%E7%9B%98/%E6%9C%89%E9%81%93/10.0.9.0/resultui/html/index.html#/javascript:;)**

**[Supplementary](D:/u%E7%9B%98/%E6%9C%89%E9%81%93/10.0.9.0/resultui/html/index.html#/javascript:;) Methods**

**Sample preparation**

**UPLC-MS analysis**

**Metabolites identified**

**[Supplementary Tables](D:/u%E7%9B%98/%E6%9C%89%E9%81%93/10.0.9.0/resultui/html/index.html#/javascript:;)**

**Supplementary Table 1.** Clinical characteristics of the participants in the discovery and validation cohorts

**Supplementary Table 2.** Clinical characteristics of the participants in the external validation cohort between responsers and non-responsers

**Supplementary Table 3.** Mixed linear model of important metabolites after adjusting for age, sex, BMI, and baseline MADRS score.

**Supplementary Table 4.** Mixed linear model of important metabolites after adjusting for more covariates.

**Supplementary Table 5.** Spearman correlation of important metabolites.

**Supplementary Table 6.** Assoication of the rate of MADRS score with important metabolites change in ketamine treatment by using partial correlation.

**Supplementary Table 7.** Thyroid function expression difference in the validation cohort.

**Supplementary Table 8.** Assoication of the rate of MADRS score with pre-treatment thyroid function in the validation cohort by using partial correlation in patients with complete ketamine treatment.

**[Supplementary](D:/u%E7%9B%98/%E6%9C%89%E9%81%93/10.0.9.0/resultui/html/index.html#/javascript:;) Methods**

**Sample preparation**

To decrease the likelihood of any prejudicial impact, the study was carried out by one investigator. Following this, the specimens were transported and ultimately subjected to ultra performance liquid chromatography-tandem mass spectrometry (UPLC-MS) analysis within one day by the Shanghai iProteome Biotechnology Co. Ltd. (Shanghai, China). Plasma samples were defrosted at a temperature of 4°C, and 50 μL portions were combined with 200 μL of chilled methanol/acetonitrile/water (2:2:1, v/v), and agitated for 30 seconds utilizing vortex technology. Thereafter, the mixture was incubated at a temperature of -20°C for a duration of 60 minutes allowing for protein removal. The resultant solution was subjected to centrifugation (10 minutes, 14,000 g, 4°C), leading to the deposition of the supernatant, which was then transferred into a new Eppendorf tube, vacuum-dried at ambient temperature and subsequently stored at a temperature of -80°C. Total peak area was used for normalization, which set the total sum of signals to a constant value for each sample [1]. Meanwhile, a pooled mixture of aliquots from all samples was prepared as a quality control (QC) sample for external standard. QC injections were used to evaluate the consistency, reproducibility, and dynamic range of data.

**UPLC-MS analysis**

Untargeted metabolomics analysis utilizing ultra performance liquid chromatography-tandem mass spectrometry (UPLC-MS) was performed using the Shimadzu Nexera UHPLC LC-30A (LC-30A Prominence, Kyoto, Japan) in combination with a Q Exactive HF-X mass spectrometer (Thermo Fisher Scientific, New York, USA). The LC-MS method involved hydrophilic interaction chromatography (HILIC) coupled to the Q Exactive HF-X mass spectrometer. Chromatographic separation was accomplished through the use of ACQUITY UPLC BEH Amide 1.7 µm (2.1 × 100 mm) columns for both negative and positive modes analysis.

The employed chromatography setup encompassed the utilization of a binary solvent system, which was propagated as a gradient of Solvent A (constituting of 10 mM ammonium acetate in water, 0.1% formic acid, 5% acetonitrile) and Solvent B (consisting of 10 mM ammonium acetate in water, 0.1% formic acid, 50% acetonitrile) so as to facilitate positive ionization mode. In negative ionization mode, either Solvent C or Solvent D was prepared by means of altering the pH of Solvent A or Solvent B through the use of ammonia. The chromatographic process was initiated either in positive or negative ionization mode, with a gradient elution of 2-98% buffer B (D) applied over a period of 12 minutes, followed by a 4-minute maintenance of the same gradient, and eventual restoration of gradient equilibrium to 2% B (D) for a duration of about 0.1 minutes, post which continuous elution for 1.9 minutes was maintained. The overall flow rate was set at 300 μL/min.

For mass spectrometric detection, both positive and negative ionization modes were applied utilizing a Q Exactive HF-X system. The MS conditions were as follows: scan range 70–1050 m/z; MSI (primary mass resolution) 120,000, AGC (automatic gain control) target 3e6, maximum IT 100 ms (positive ionization mode) and 200 ms (negative ionization mode); secondary mass resolution 7,500, AGC target 2e5, maximum IT 50 ms.

The mass spectrometry (MS) settings employed included a scan range of 70-1050 m/z; with MSI (primary mass resolution) at 120,000, utilizing an AGC (automatic gain control) target of 3e6, with a maximum IT of 100 ms (positive ionization mode) and 200 ms (negative ionization mode). Moreover, secondary mass resolution of 7,500 was adopted, with an AGC target set at 2e5, and a maximum IT of 50 ms. The analysis involved the use of HCD fragmentation mode, where normalized collision energy was set at 20, 40, and 60; with isolation window being 1.5 m/z, and a sub-ion scanning range of 200-2000 m/z.

**Metabolites identified**

The Compound Discoverer program (version 3.1) was utilized for compound annotation of the UPLC-MS data, with references being derived from multiple databases, such as the mzCloud database (http://www.mzCloud.org/), HMDB database (http://www.hmdb.ca/), LipidMAPS database (http://www.lipidmaps.org/), and ChemSpider database (http://www.chemspider.com/), among others. Raw instrument data (.RAW) were acquired by Xcalibur software (Thermo Fisher Scientific) and exported to Compound Discoverer 3.1 (CD3.1) for deconvolution, alignment, and annotation. For peak picking, the following parameters were used: The frame time width was 2 min, centWave m/z < 5 ppm, with MS2 matched. The intensity threshold for component extraction was 1e6, the maximum retention time shift for peak alignment was 0.2 min and signal-to-noise threshold was 3. Apply Intensity Threshold was used, which specifies whether to apply an automatic intensity threshold that sets the threshold intensity by calculating the spectrum noise level. In the extracted ion features, only the variables having more than 50% of the nonzero measurement values in at least one group were kept. Compound identification of metabolites by MS/MS spectra with an in-house database established with available authentic standards.

Identical metabolites occurring in both negative and positive ion modes were combined and subsequently averaged. Subsequent to the analysis, a total of 647 endogenous metabolites were shortlisted, and their properties were characterized utilizing the Kyoto Encyclopedia of Genes and Genomes (KEGG, https://www.kegg.jp/) and the Pubchem database (https://pubchem.ncbi.nlm.nih.gov/).

**References**

1. Blaise BJ, Correia GDS, Haggart GA, Surowiec I, Sands C, Lewis MR, Pearce JTM, Trygg J, Nicholson JK, Holmes E, Ebbels TMD: Statistical analysis in metabolic phenotyping. Nat Protoc 2021, 16:4299-4326.

**Supplementary Table 1.** Clinical characteristics of the participants in the discovery and validation cohorts

| **Characteristic** | **Discovery cohort**  **(n=40)** | **Validation cohort**  **(n=24)** | **Statistics**  **p** |
| --- | --- | --- | --- |
| Sex, Male, n (%)^a^ | 16 (40.0%) | 11 (45.8%) | 0.845 |
| Age (years), Median (IQR)^b^ | 33 (24-46) | 30 (24-40) | 0.437 |
| BMI, Mean (SD)^c^ | 21.93 (3.48) | 21.83 (3.43) | 0.916 |
| Education (years), Median (IQR)^b^ | 12 (9-15) | 14 (12-15) | 0.510 |
| Onset of age (years), Median (IQR)^b^ | 24 (19-35) | 23 (18-31) | 0.232 |
| Duration (months), Median (IQR)^b^ | 51 (23-111) | 78 (24-144) | 0.409 |
| Smoking, Smoker, n (%)^a^ | 5 (12.5%) | 7 (29.2%) | 0.113 |
| Baseline MADRS score, Mean (SD)^c^ | 31.80 (6.50) | 32.83 (6.36) | 0.535 |
| MADRS Reduction, Responders, n (%)^a^ | 23 (57.5%) | 17 (70.8%) | 0.424 |
| Current antidepressant medications |  |  |  |
| Fluoxetine equivalents, mg, Mean (SD)^c^ | 41.12 (23.30) | 33.27 (19.72) | 0.166 |
| Antipsychotics, n (%)^a^ | 27 (67.5%) | 11 (45.8%) | 0.088 |
| Benodiazepine, n (%)^a^ | 22 (55.0%) | 4 (16.7%) | **0.003** |
| Emotional stabilizer, n (%)^a^ | 7 (17.5%) | 5 (20.8%) | 0.741 |

^a^Analyzed by the Chi-square test.

^b^Analyzed by Mann-Whitney U test.

^c^Analyzed by Independent two-sample t-test.

**Supplementary Table 2.** Clinical characteristics of the participants in the external validation cohort between responsers and non-responsers.

| **Characteristic** | **Non-responser**  **(n=12)** | **Responser**  **(n=12)** | **Statistics**  **p** |
| --- | --- | --- | --- |
| Sex, Male, n (%)^a^ | 7 (58.3%) | 4 (33.3%) | 0.413 |
| Age (years), Median (IQR)^b^ | 28 (19-40) | 34 (24-38) | 0.623 |
| BMI, Mean (SD)^c^ | 21.35 (2.35) | 22.32 (4.32) | 0.503 |
| Education (years), Median (IQR)^b^ | 12 (9-14) | 15 (14-16) | **0.012** |
| Onset of age (years), Median (IQR)^b^ | 20 (17-28) | 23 (21-31) | 0.386 |
| Duration (months), Median (IQR)^b^ | 84 (21-160) | 78 (26-108) | 0.977 |
| Smoking, Smoker, n (%)^a^ | 4 (33.3%) | 3 (25.9%) | 1.000 |
| Baseline MADRS score, Mean (SD)^c^ | 33.25 (6.55) | 32.42 (6.42) | 0.756 |
| Post-treatment MADRS score, Mean (SD)^c^ | 27.42 (7.54) | 10.17 (5.32) | **<0.001** |
| Current antidepressant medications |  |  |  |
| Fluoxetine equivalents, mg, Mean (SD)^c^ | 29.55 (19.81) | 37.00 (19.85) | 0.388 |
| Antipsychotics, n (%)^a^ | 5 (41.7%) | 6 (50.0%) | 1.000 |
| Benodiazepine, n (%)^a^ | 3 (25.0%) | 1 (8.3%) | 0.590 |
| Emotional stabilizer, n (%)^a^ | 3 (25.0%) | 2 (16.7%) | 1.000 |

^a^Analyzed by the Chi-square test.

^b^Analyzed by Mann-Whitney U test.

^c^Analyzed by Independent two-sample t-test.

**Supplementary Table 3.** Mixed linear model of important metabolites after adjusting for age, sex, BMI, and baseline MADRS score.

| **Metabolites** | **Group Effect** | | **Time Effect** | | **Interaction Effect** | | |
| --- | --- | --- | --- | --- | --- | --- | --- |
|  | **Beta (SE)** | **P value**^a^ | **Beta (SE)** | **P value**^a^ | **Beta (SE)** | **F value** | **P value**^a^ |
| 4-Guanidinobutanoic acid | -0.440 (0.271) | 0.226 | 0.004 (0.207) | 0.984 | 0.587 (0.273) | 4.607 | **0.040** |
| ADP | -0.789 (0.562) | 0.304 | 0.928 (0.515) | 0.096 | 1.566 (0.680) | 5.308 | **0.040** |
| Adrenic acid | -0.418 (0.518) | 0.510 | -2.817 (0.528) | **0.000** | 1.638 (0.696) | 5.535 | **0.040** |
| Oxoglutaric acid | -0.164 (0.749) | 0.828 | -1.032 (0.559) | 0.096 | 1.644 (0.737) | 4.980 | **0.040** |
| (3Z)-Phycocyanobilin | -0.250 (0.210) | 0.365 | -0.479 (0.194) | **0.032** | 0.587 (0.255) | 5.286 | **0.040** |
| Linoleic acid | -0.128 (0.255) | 0.696 | -1.537 (0.257) | **0.000** | 0.812 (0.339) | 5.737 | **0.040** |
| Arachidonic acid | -0.164 (0.203) | 0.510 | -1.179 (0.144) | **0.000** | 0.509 (0.190) | 7.154 | **0.040** |
| Myristic acid | -0.072 (0.212) | 0.778 | -1.329 (0.210) | **0.000** | 0.677 (0.277) | 5.964 | **0.040** |
| L-Histidine | -0.170 (0.101) | 0.225 | -0.240 (0.106) | **0.049** | 0.318 (0.140) | 5.145 | **0.040** |
| D-Serine | -0.143 (0.136) | 0.417 | 0.064 (0.091) | 0.518 | -0.248 (0.121) | 4.246 | **0.046** |
| Nicotinuric acid | 0.619 (0.213) | 0.054 | 1.374 (0.207) | **0.000** | -0.594 (0.272) | 4.752 | **0.040** |
| 5-(2-Hydroxyethyl)-4-methylthiazole | 1.018 (0.806) | 0.352 | 1.797 (0.851) | 0.062 | -2.731 (1.122) | 5.926 | **0.040** |
| (R)-2,3-Dihydroxy-3-methylvalerate | 0.160 (0.068) | 0.122 | 0.313 (0.068) | **0.000** | -0.282 (0.089) | 10.034 | **0.027** |
| DEHP | 2.951 (0.890) | **0.036** | 1.912 (0.774) | **0.032** | -2.977 (1.021) | 8.495 | **0.027** |
| Biliverdin | 0.938 (0.485) | 0.220 | 1.517 (0.473) | **0.007** | -1.884 (0.624) | 9.123 | **0.027** |
| Tungstate | 0.199 (0.112) | 0.225 | 0.464 (0.104) | **0.000** | -0.303 (0.138) | 4.850 | **0.040** |
| Bilirubin | 1.358 (0.588) | 0.122 | 0.879 (0.620) | 0.185 | -1.931 (0.818) | 5.574 | **0.040** |
| (R)-4-Dehydropantoate | 0.453 (0.263) | 0.225 | 0.404 (0.221) | 0.096 | -0.865 (0.292) | 8.788 | **0.027** |

The mixed linear model was used to assess the association between MADRS score and baseline thyroid levels, accounting for fixed effects of group, time-point, group*time-point interaction, and adjusting for sex, age, BMI, and baseline MADRS score, with a random intercept for each individual.

^a^ P-values were adjusted by using the Benjamini–Hochberg procedure.

Abbreviations: ADP, Adenosine diphosphate; DEHP, Bis(2-ethylhexyl) phthalate.

**Supplementary Table 4.** Mixed linear model of important metabolites after adjusting for more covariates.

| **Metabolites**^a^ | **Group Effect** | | **Time Effect** | | **Interaction Effect** | | |
| --- | --- | --- | --- | --- | --- | --- | --- |
|  | **Beta (SE)** | **P value**^a^ | **Beta (SE)** | **P value**^a^ | **Beta (SE)** | **F value** | **P value**^a^ |
| 4-Guanidinobutanoic acid | -0.413 (0.289) | 0.326 | 0.004 (0.207) | 0.984 | 0.587 (0.273) | 1.314 | **0.040** |
| ADP | -0.690 (0.536) | 0.374 | 0.928 (0.515) | 0.096 | 1.566 (0.680) | 6.248 | **0.040** |
| Adrenic acid | -0.244 (0.505) | 0.712 | -2.817 (0.519) | **0.000** | 1.638 (0.684) | 0.985 | **0.040** |
| Oxoglutaric acid | -0.298 (0.810) | 0.716 | -1.032 (0.559) | 0.096 | 1.644 (0.737) | 0.295 | **0.040** |
| (3Z)-Phycocyanobilin | -0.103 (0.181) | 0.712 | -0.479 (0.185) | **0.028** | 0.587 (0.244) | 0.536 | **0.040** |
| Linoleic acid | -0.114 (0.269) | 0.715 | -1.537 (0.257) | **0.000** | 0.812 (0.339) | 0.511 | **0.040** |
| Arachidonic acid | -0.194 (0.195) | 0.451 | -1.179 (0.144) | **0.000** | 0.509 (0.190) | 3.175 | **0.040** |
| Myristic acid | -0.114 (0.219) | 0.712 | -1.329 (0.210) | **0.000** | 0.677 (0.277) | 1.366 | **0.040** |
| L-Histidine | -0.172 (0.107) | 0.266 | -0.240 (0.110) | 0.057 | 0.318 (0.144) | 0.226 | **0.040** |
| D-Serine | -0.157 (0.145) | 0.435 | 0.064 (0.091) | 0.518 | -0.248 (0.121) | 0.082 | **0.046** |
| Nicotinuric acid | 0.570 (0.219) | 0.126 | 1.374 (0.207) | **0.000** | -0.594 (0.272) | 1.129 | **0.040** |
| 5-(2-Hydroxyethyl)-4-methylthiazole | 1.007 (0.854) | 0.406 | 1.797 (0.877) | 0.071 | -2.731 (1.156) | 0.567 | **0.040** |
| (R)-2,3-Dihydroxy-3-methylvalerate | 0.156 (0.069) | 0.185 | 0.313 (0.068) | **0.000** | -0.282 (0.089) | 0.022 | **0.027** |
| DEHP | 2.893 (0.964) | 0.090 | 1.912 (0.774) | **0.032** | -2.977 (1.021) | 0.059 | **0.027** |
| Biliverdin | 0.906 (0.521) | 0.266 | 1.517 (0.473) | **0.007** | -1.884 (0.624) | 0.278 | **0.027** |
| Tungstate | 0.211 (0.121) | 0.266 | 0.464 (0.104) | **0.000** | -0.303 (0.138) | 0.505 | **0.040** |
| Bilirubin | 1.325 (0.619) | 0.185 | 0.879 (0.635) | 0.196 | -1.931 (0.837) | 0.837 | **0.040** |
| (R)-4-Dehydropantoate | 0.442 (0.263) | 0.266 | 0.404 (0.221) | 0.096 | -0.865 (0.292) | 0.561 | **0.027** |

The mixed linear model was used to assess the association between MADRS score and baseline thyroid levels, accounting for fixed effects of group, time-point, group*time-point interaction, and adjusting for sex, age, BMI, baseline MADRS score, duration, fluoxetine equivalents, smoking status, alcohol consumption, and onset of age, with a random intercept for each individual.

^a^ P-values were adjusted by using the Benjamini–Hochberg procedure.

Abbreviations: ADP, Adenosine diphosphate; DEHP, Bis(2-ethylhexyl) phthalate.

**Supplementary Table 5.** Spearman correlation analysis of between important metabolites and MADRS scores.

| **Metabolites** | **HMDB** | **PubChem** | **Rho** | **P value** | **Q value**^a^ |
| --- | --- | --- | --- | --- | --- |
| 4-Guanidinobutanoic acid | HMDB0003464 | 500 | 0.545 | **0.000** | **0.000** |
| ADP | HMDB0001341 | 6022 | 0.481 | **0.002** | **0.018** |
| Adrenic acid | HMDB0002226 | 5497181 | 0.433 | **0.005** | **0.023** |
| Oxoglutaric acid | HMDB0000208 | 51 | 0.409 | **0.009** | **0.026** |
| (3Z)-Phycocyanobilin | METPA0687 | NA | 0.366 | **0.020** | **0.032** |
| Linoleic acid | HMDB0000673 | 5280450 | 0.355 | **0.024** | **0.032** |
| Arachidonic acid | HMDB0001043 | 444899 | 0.349 | **0.027** | **0.032** |
| Myristic acid | HMDB0000806 | 11005 | 0.349 | **0.027** | **0.032** |
| L-Histidine | HMDB0000177 | 6274 | 0.329 | **0.038** | **0.038** |
| D-Serine | HMDB0003406 | 71077 | -0.330 | **0.038** | **0.038** |
| Nicotinuric acid | HMDB0003269 | 68499 | -0.334 | **0.035** | **0.038** |
| 5-(2-Hydroxyethyl)-4-methylthiazole | HMDB0032985 | 1136 | -0.361 | **0.022** | **0.032** |
| (R)-2,3-Dihydroxy-3-methylvalerate | HMDB0012140 | 448154 | -0.365 | **0.021** | **0.032** |
| DEHP | NA | NA | -0.375 | **0.017** | **0.032** |
| Biliverdin | HMDB0001008 | 5353439 | -0.383 | **0.015** | **0.032** |
| Tungstate | NA | NA | -0.404 | **0.010** | **0.026** |
| Bilirubin | HMDB0000054 | 21252250 | -0.405 | **0.010** | **0.026** |
| (R)-4-Dehydropantoate | METPA0117 | NA | -0.446 | **0.004** | **0.023** |

^a^ P-values were adjusted by using the Benjamini-Hochberg procedure.

Abbreviations: ADP, Adenosine diphosphate; DEHP, Bis(2-ethylhexyl) phthalate.

**Supplementary Table 6.** Assoication of the rate of MADRS score with important metabolites change in ketamine treatment by using partial correlation.

| **Metabolites** | **Crude Model** ^a^ | | **Model 1**^b^ | | **Model 2**^c^ | |
| --- | --- | --- | --- | --- | --- | --- |
|  | **Cor** | **P value**^d^ | **Cor** | **P value**^d^ | **Cor** | **P value**^d^ |
| 4-Guanidinobutanoic acid | 0.562 | **<0.001** | 0.563 | **0.006** | 0.580 | **0.011** |
| ADP | 0.472 | **0.012** | 0.433 | **0.034** | 0.479 | **0.024** |
| Adrenic acid | 0.262 | 0.132 | 0.210 | 0.232 | 0.215 | 0.260 |
| Oxoglutaric acid | 0.378 | **0.032** | 0.373 | 0.054 | 0.329 | 0.116 |
| (3Z)-Phycocyanobilin | 0.291 | 0.096 | 0.292 | 0.117 | 0.335 | 0.116 |
| Linoleic acid | 0.248 | 0.133 | 0.237 | 0.184 | 0.295 | 0.149 |
| Arachidonic acid | 0.246 | 0.133 | 0.269 | 0.145 | 0.278 | 0.166 |
| Myristic acid | -0.035 | 0.831 | -0.021 | 0.902 | -0.016 | 0.932 |
| L-Histidine | 0.302 | 0.087 | 0.293 | 0.117 | 0.242 | 0.228 |
| D-Serine | -0.401 | **0.029** | -0.426 | **0.034** | -0.410 | **0.049** |
| Nicotinuric acid | -0.315 | 0.085 | -0.257 | 0.157 | -0.231 | 0.238 |
| 5-(2-Hydroxyethyl)-4-methylthiazole | -0.253 | 0.133 | -0.366 | 0.054 | -0.413 | **0.049** |
| (R)-2,3-Dihydroxy-3-methylvalerate | -0.308 | 0.087 | -0.362 | 0.054 | -0.462 | **0.027** |
| DEHP | -0.389 | **0.029** | -0.382 | 0.054 | -0.353 | 0.102 |
| Biliverdin | -0.416 | **0.029** | -0.368 | 0.054 | -0.512 | **0.020** |
| Tungstate | -0.426 | **0.027** | -0.505 | **0.015** | -0.523 | **0.020** |
| Bilirubin | -0.394 | **0.029** | -0.307 | 0.112 | -0.297 | 0.149 |
| (R)-4-Dehydropantoate | -0.509 | **0.009** | -0.477 | **0.019** | -0.477 | **0.024** |

^a^ The partial correlation was used to assess the association between MADRS reduction and metabolites changes (log₂-transformed).

^b^ In model 2, we additionally adjusted for sex, age, BMI, baseline MADRS score in the partial correlation.

^c^ In model 3, we additionally adjusted for sex, age, BMI, baseline MADRS score, duration, fluoxetine equivalents, smoking status, alcohol consumption, and onset of age in the partial correlation.

^d^ P-values were adjusted by using the Benjamini–Hochberg procedure.

**Supplementary Table 7.** Thyroid function expression difference in the validation cohort.

| **Variables** | **Total**  **(N=24)** | **Non-responders**  **(N = 12)** | **Responders**  **(N = 12)** | **Statistics**  **p** |
| --- | --- | --- | --- | --- |
| TSH, mIU/L | 1.69 (0.91) | 1.59 (1.05) | 1.79 (0.78) | 0.591 |
| FT3, pmol/L | 4.03 (0.51) | 4.16 (0.63) | 3.91 (0.33) | 0.240 |
| FT4, pmol/L | 12.66 (1.74) | 12.93 (1.94) | 12.39 (1.56) | 0.459 |
| TT3, nmol/L | 1.34 (0.25) | 1.37 (0.33) | 1.30 (0.13) | 0.538 |
| TT4, nmol/L | 83.71 (22.71) | 85.78 (27.04) | 81.64 (18.37) | 0.666 |

Data were presented as mean (SD).

The p-value was calculated by comparing non-responders and responders.

Abbreviations: FT3, free triiodothyronine; FT4, free thyroxine; TT3, total triiodothyronine; TT4, total thyroxine; TSH, thyroid-stimulating hormone.

**Supplementary** **Table 8.** Assoication of the rate of MADRS score with pre-treatment thyroid function in the validation cohort by using partial correlation in patients with complete ketamine treatment.

| **Variables** | **Model 1**^a^ | | **Model 2**^b^ | |
| --- | --- | --- | --- | --- |
|  | **Cor** | **P value** | **Cor** | **P value** |
| TSH, mIU/L | -0.230 | 0.429 | -0.109 | 0.781 |
| FT3, pmol/L | -0.353 | 0.215 | -0.681 | **0.043** |
| FT4, pmol/L | -0.014 | 0.963 | -0.007 | 0.986 |
| TT3, nmol/L | -0.395 | 0.162 | -0.657 | 0.055 |
| TT4, nmol/L | -0.196 | 0.501 | -0.268 | 0.485 |

The partial correlation was used to assess the association between MADRS reduction and metabolites changes (log₂-transformed).

^a^ In model 1, we additionally adjusted for sex, age, BMI, baseline MADRS score in the partial correlation.

^b^ In model 2, we additionally adjusted for sex, age, BMI, baseline MADRS score, duration, fluoxetine equivalents, smoking status, alcohol consumption, and onset of age in the partial correlation.

Abbreviations: FT3, free triiodothyronine; FT4, free thyroxine; TT3, total triiodothyronine; TT4, total thyroxine; TSH, thyroid-stimulating hormone.
